# Genetically predicted ZDHHC8 expression and osteoporosis risk: A two-sample Mendelian randomization and metabolite mediation study

**DOI:** 10.1097/MD.0000000000050859

**Published:** 2026-09-25

**Authors:** Wenli Yang, Kaiwen Yang, Shangyi Xu, Shuang Yang

**Affiliations:** a Health Management Department, Luzhou People’s Hospital, Luzhou, Sichuan, China; b Department of Radiology, The First Affiliated Hospital of Chongqing College of Chinese Medicine, Chongqing, China.

**Keywords:** eQTL, Mendelian randomization, metabolite mediation, osteoporosis, S-palmitoylation, ZDHHC8

## Abstract

Protein S-palmitoylation regulates protein localization, stability, and signaling and has emerging roles in bone-cell biology. We investigated whether genetically predicted expression of palmitoylation-related genes is associated with osteoporosis risk and explored circulating metabolites as candidate mediators. We performed a 2-sample Mendelian randomization (MR) study using cis-expression quantitative trait locus instruments for 22 palmitoylation-related genes, FinnGen R10 osteoporosis data (8017 cases and 391,037 controls), and 1400 circulating metabolite traits (1091 metabolites and 309 metabolite ratios). Inverse-variance weighted was the primary estimator. Weighted-median and MR-Egger analyses, Cochran *Q* testing, and instrument-strength assessment were used as sensitivity analyses. Summary-data-based MR with the heterogeneity in dependent instruments test was used as an additional gene expression validation. Gene Expression Omnibus bone marrow mesenchymal stem cell datasets were used for expression comparison; GSE35959 was treated as the SuperSeries containing GSE35956 and GSE35958 rather than as an independent replication cohort. Two-step MR and the product-of-coefficients approach were used to estimate candidate mediation effects. Genetically predicted zinc finger DHHC-type palmitoyltransferase 8 (ZDHHC8) expression was associated with higher osteoporosis risk (odds ratio = 1.23, 95% confidence interval: 1.09–1.39, *P* = .0007), and the association met the Bonferroni threshold for the 22-gene screen. Summary-data-based MR provided additional, nominal evidence for ZDHHC8 (*P* = .0415). ZDHHC8 expression was higher in osteoporosis-derived bone marrow mesenchymal stem cells in the Gene Expression Omnibus comparison. Three metabolite traits: 3-(3-amino-3-carboxypropyl)uridine (acp3U), 2-hydroxyhippurate, and X-25343, showed directionally coherent 2-step MR estimates, with estimated mediated proportions of 11%, 9%, and 10%, respectively. Because these metabolite findings did not meet a Bonferroni threshold for 1400 tests, they are interpreted as exploratory candidate mediators. Genetically predicted ZDHHC8 expression is associated with osteoporosis risk across complementary genetic and expression analyses. The metabolite results suggest possible intermediate pathways but remain hypothesis-generating. These findings support further functional study of ZDHHC8 in bone cells rather than immediate clinical targeting.

## 1. Introduction

Osteoporosis is a systemic skeletal disease characterized by low bone mass, impaired bone microarchitecture, and increased fracture risk.^[1,2]^ Its burden rises with population aging and differs by sex, age, ancestry, hormonal status, lifestyle, and comorbid disease.^[3–5]^ Current treatments reduce fracture risk, but response, adherence, adverse effects, and residual fracture risk remain important clinical problems. Identifying molecular pathways that contribute to bone remodeling may therefore provide new targets for mechanistic and translational research.

Protein S-palmitoylation is a reversible posttranslational modification in which a 16-carbon palmitoyl group is attached mainly to cysteine residues through a thioester bond.^[6–8]^ This modification can alter membrane localization, trafficking, stability, and signaling. It is catalyzed by zinc finger Aspartate–Histidine–Histidine–Cysteine (DHHC)-domain-containing S-acyltransferases and reversed by depalmitoylating enzymes.^[8–10]^ Palmitoylation is well studied in cancer, neurological disease, and immune signaling, but its role in bone metabolism is less defined.

Several observations support studying palmitoylation in osteoporosis. Wnt ligand acylation is required for normal Wnt secretion and signaling, and Wnt/-catenin signaling is central to osteoblast differentiation and bone formation.^[11–13]^ In addition, experimental inhibition of protein S-palmitoylation can alter osteoblast and osteoclast differentiation and bone remodeling.^[14,15]^ These findings suggest that palmitoylation-related enzymes may influence osteoporosis, although the relevant enzymes and causal direction remain uncertain.

Systemic metabolism is also closely linked to skeletal homeostasis.^[16–18]^ Circulating amino acids, lipids, nucleotides, and other small molecules may reflect or participate in pathways that affect osteoblasts, osteoclasts, and the bone marrow microenvironment. Conventional observational metabolomics, however, can be affected by age, sex, body composition, renal function, medication use, diet, physical activity, smoking, and reverse causation. This makes it difficult to determine whether a metabolite is a cause, consequence, or correlate of osteoporosis.

Mendelian randomization (MR) uses genetic variants associated with an exposure as instrumental variable (IV)s. Under the relevance, independence, and exclusion-restriction assumptions, genetic allocation can reduce bias from conventional confounding and reverse causation.^[19,20]^ A 2-sample design also allows large, independent genome-wide association study (GWAS) resources to be combined efficiently. This approach is useful here because gene expression and circulating metabolites are both vulnerable to disease-related changes in conventional observational studies. We therefore used cis-expression quantitative trait locus (eQTL)-based MR to screen a prespecified set of palmitoylation-related genes, followed by summary-data-based MR (SMR), bone marrow mesenchymal stem cell (BMSC) expression analysis, and exploratory 2-step MR of circulating metabolites. The aim was not to prove that zinc finger DHHC-type palmitoyltransferase 8 (ZDHHC8) is already a drug target, but to prioritize a gene-metabolite axis for further experimental study.

## 2. Method

This study used a 2-sample MR framework to assess the association between genetically predicted expression of palmitoylation-related genes and osteoporosis. Circulating metabolite traits were then evaluated as potential intermediate factors using 2-step MR. Public summary-level eQTL and GWAS data were used, followed by SMR and Gene Expression Omnibus (GEO)-based expression assessment. The analytical workflow is shown in Figure 1. We followed the principles of Strengthening the Reporting of Observational Studies in Epidemiology–MR for transparent reporting.^[20]^

**Figure 1.**
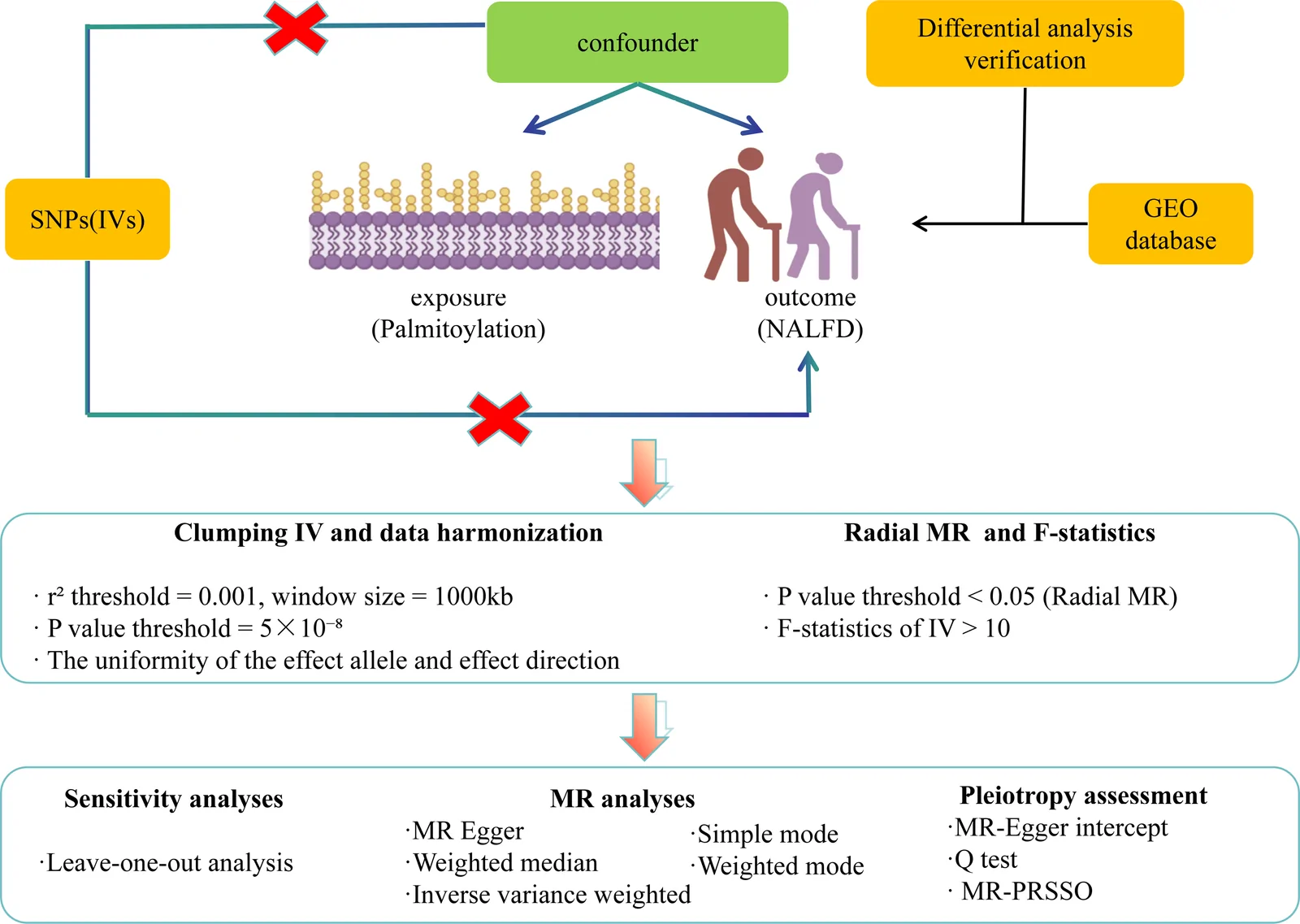
Study workflow for the 2-sample MR, SMR/HEIDI, GEO expression analysis, and exploratory metabolite mediation analysis. MR reduces conventional confounding and reverse causation only when the IV assumptions are satisfied; residual bias from pleiotropy, population structure, and measurement error remains possible. GEO = Gene Expression Omnibus, HEIDI = heterogeneity in dependent instruments, IV = instrumental variable, MR = Mendelian randomization, MR-PRESSO = Mendelian Randomization Pleiotropy RESidual Sum and Outlier, NAFLD = nonalcoholic fatty liver disease, SMR = summary-data-based Mendelian randomization, SNP = single-nucleotide polymorphism.

### 2.1. Sources of data

#### 2.1.1. GWAS data of palmitoylated genes

The eQTL data were obtained from the eQTLGen consortium.^[21]^ We focused on cis-eQTLs from blood because cis variants are generally more proximal to gene expression than trans-eQTLs and may reduce, although not eliminate, horizontal pleiotropy. A list of 31 palmitoylation-related genes was compiled from the published literature^[6,22,23]^ before testing osteoporosis associations. This list was intersected with genes available in eQTLGen, leaving 22 genes for analysis. Thus, genes were not selected according to their osteoporosis *P* values. Instrument selection and strength criteria are described in Section 2.2.

#### 2.1.2. Serum metabolite data

Circulating metabolite GWAS summary statistics were obtained from the genomic atlas of the plasma metabolome.^[24]^ We did not manually select individual metabolites. Instead, all 1400 traits available in that data resource were screened: 1091 plasma metabolites and 309 metabolite ratios. Among the 1091 metabolites, 850 were assigned to major biochemical classes, including lipids, amino acids, xenobiotics, nucleotides, cofactors and vitamins, carbohydrates, peptides, and energy-related metabolites; 241 were not chemically identified. This complete-panel strategy reduced selective reporting at the metabolite-selection stage, but it also created a substantial multiple-testing burden that was considered in interpretation.

#### 2.1.3. Osteoporosis GWAS data

Osteoporosis GWAS summary statistics were obtained from FinnGen R10 (phenocode M13OSTEOPOROSIS), including 8017 cases and 391,037 controls of European ancestry. The FinnGen endpoint is registry-based and uses osteoporosis diagnosis codes from hospital discharge and cause-of-death records (including International Classification of Diseases-10 M80-M82, with corresponding earlier dual-energy X-ray absorptiometry [DXA] codes), rather than a single uniform DXA-derived bone mineral density threshold. This broad clinical phenotype increases sample size but may introduce outcome misclassification. The summary statistics are publicly available from FinnGen.

#### 2.1.4. GEO dataset

GEO data were used as a tissue-relevant expression check in human BMSCs. GSE35958 contains 5 elderly women with primary osteoporosis and 4 age-matched non-osteoporotic elderly controls. GSE35956 contains the same 5 osteoporosis donors and 5 middle-aged non-osteoporotic controls. Both use the GPL570 platform. GSE35959 is the SuperSeries that contains these SubSeries and therefore was not treated as a third independent validation dataset. This distinction is important because the osteoporosis samples overlap. GSE35958 provides the less age-confounded comparison, whereas GSE35956 is more vulnerable to confounding by donor age. The GEO analysis was therefore used as supportive expression evidence, not as independent causal validation.

### 2.2. Selection of IVs

IVs were selected to satisfy the MR relevance assumption. We first considered variants at *P* < 5 × 10^−8^. When too few variants were available for a gene, a relaxed threshold of *P* < 5 × 10^−6^ was used to retain analyzable cis-eQTL instruments; this decision increases power but also raises the risk of weak-instrument bias. Variants were clumped at *R* < 0.1 within 10,000 kb. Proxy/fallback variants and single-nucleotide polymorphism (SNP)s with *F* statistics < 10 were excluded. The F statistic was used to assess instrument strength, and the retained SNPs are listed in Supplementary Material 1, Supplemental Digital Content 1. Effect alleles were harmonized between exposure and outcome datasets before MR estimation.

### 2.3. MR analyses and SMR validation

Analyses were performed using R software (version 4.2.1; R Foundation for Statistical Computing) with the TwoSampleMR package (version 0.5.6). Inverse-variance weighted (IVW) was used as the primary MR estimator when more than 1 instrument was available. IVW provides an efficient estimate when the instruments are valid or when horizontal pleiotropic effects are balanced. The weighted-median estimator and MR-Egger regression were used as sensitivity methods because they rely on different assumptions about invalid instruments.^[25,26]^ MR estimates for osteoporosis were reported as odds ratio (OR)s with 95% confidence interval (CI)s per genetically predicted increase in gene expression or metabolite level, according to the scale of the source GWAS.

SMR was used as a complementary analysis integrating eQTL and osteoporosis GWAS summary statistics.^[27]^ SMR tests whether genetically predicted gene expression is associated with the outcome using the top cis-eQTL signal. The heterogeneity in dependent instruments (HEIDI) test was used to assess whether the observed association was compatible with a shared underlying variant rather than distinct variants in linkage disequilibrium. A nonsignificant HEIDI result does not prove causality; it only provides less evidence against a shared signal. SMR version 1.3.1 was run with default settings.

### 2.4. Sensitivity analyses

Sensitivity analyses assessed instrument heterogeneity and horizontal pleiotropy. Cochran *Q* was used to test heterogeneity among SNP-specific causal estimates; *P* < .05 indicates evidence that the estimates vary more than expected by sampling error. MR-Egger regression was used to obtain both a pleiotropy-robust slope and an intercept test for directional horizontal pleiotropy; an intercept *P* < .05 was considered evidence of directional pleiotropy.^[25]^ The weighted-median estimate was also examined because it can remain consistent when up to half of the total weight arises from invalid instruments.^[26]^ Concordant effect direction across IVW, weighted median, and MR-Egger was considered supportive, but nonsignificant *Q* or MR-Egger intercept tests were not interpreted as proof that pleiotropy was absent. Full sensitivity outputs are provided in Supplementary Material 3, Supplemental Digital Content 2.

### 2.5. GEO expression analysis

Normalized GEO expression data were used to compare ZDHHC8 expression in osteoporosis-derived BMSCs with the prespecified non-osteoporotic groups. GSE35958 and GSE35956 were interpreted as 2 comparison schemes derived from related samples, not as independent replication cohorts. The principal purpose of this analysis was to examine whether the direction of ZDHHC8 expression was consistent with the genetically predicted association. Because of the small sample sizes and differences in age, sex distribution, fracture status, osteoarthritis/hip dysplasia in controls, passage number, and other unmeasured clinical factors, the GEO analysis was treated as supportive observational evidence.

### 2.6. Two-step MR mediation analysis

For candidate mediation analysis, we first estimated the effect of genetically predicted ZDHHC8 expression on each metabolite and then the effect of the metabolite on osteoporosis. The indirect effect was calculated using the product-of-coefficients method (indirect = 1 × 2), and the direct effect was estimated as direct = total − indirect. The mediated proportion was calculated as indirect/total × 100%.^[28]^ All coefficients were handled on a compatible linear/log-odds scale before multiplication. This approach assumes valid instruments for both steps, no important exposure-mediator interaction on the modeled scale, and no pathway from the exposure instruments to the outcome that bypasses the modeled exposure. Because only summary data were available, mediation estimates should be interpreted as approximate and hypothesis-generating.

### 2.7. Statistical significance and multiple testing

All tests were 2-sided. For the primary screen of 22 palmitoylation-related genes, *P* < .05/22 = 2.27 × 10^−^ was considered Bonferroni-corrected evidence, while *P* < .05 was described as nominal or suggestive. The metabolite stage screened 1400 traits; the corresponding Bonferroni threshold is 3.57 × 10^−^. Reported metabolite associations with *P* < .05 that did not meet this threshold were therefore labeled exploratory candidate mediators rather than definitive findings. Statistical significance was interpreted together with effect size, 95% CI, instrument strength, sensitivity analyses, and biological plausibility.

### 2.8. Ethics and data governance

This study analyzed de-identified, publicly available summary-level genetic data and public GEO expression data. No new individual-level participant data were collected. Therefore, no new institutional review board approval or informed consent was required for this secondary analysis. Ethical approval and informed consent were obtained by the original studies according to their respective protocols. The present work does not attempt to identify individual participants.

## 3. Results

### 3.1. MR and SMR findings on palmitoylation genes and osteoporosis

The primary gene screen identified positive IVW associations for ZDHHC14 (OR = 1.09, 95% CI: 1.04–1.14, *P* = .0005), ZDHHC16 (OR = 1.39, 95% CI: 1.01–1.91, *P* = .0438), and ZDHHC8 (OR = 1.23, 95% CI: 1.09–1.39, *P* = .0007), whereas ZDHHC5 showed an inverse association (OR = 0.82, 95% CI: 0.76–0.89, *P* < .0001) (Fig. 2 and Supplementary Material 2, Supplemental Digital Content 3). After Bonferroni correction for 22 genes (*P* < 2.27 × 10^−^), ZDHHC14, ZDHHC8, and ZDHHC5 remained supported, whereas ZDHHC16 was considered nominal.

**Figure 2.**
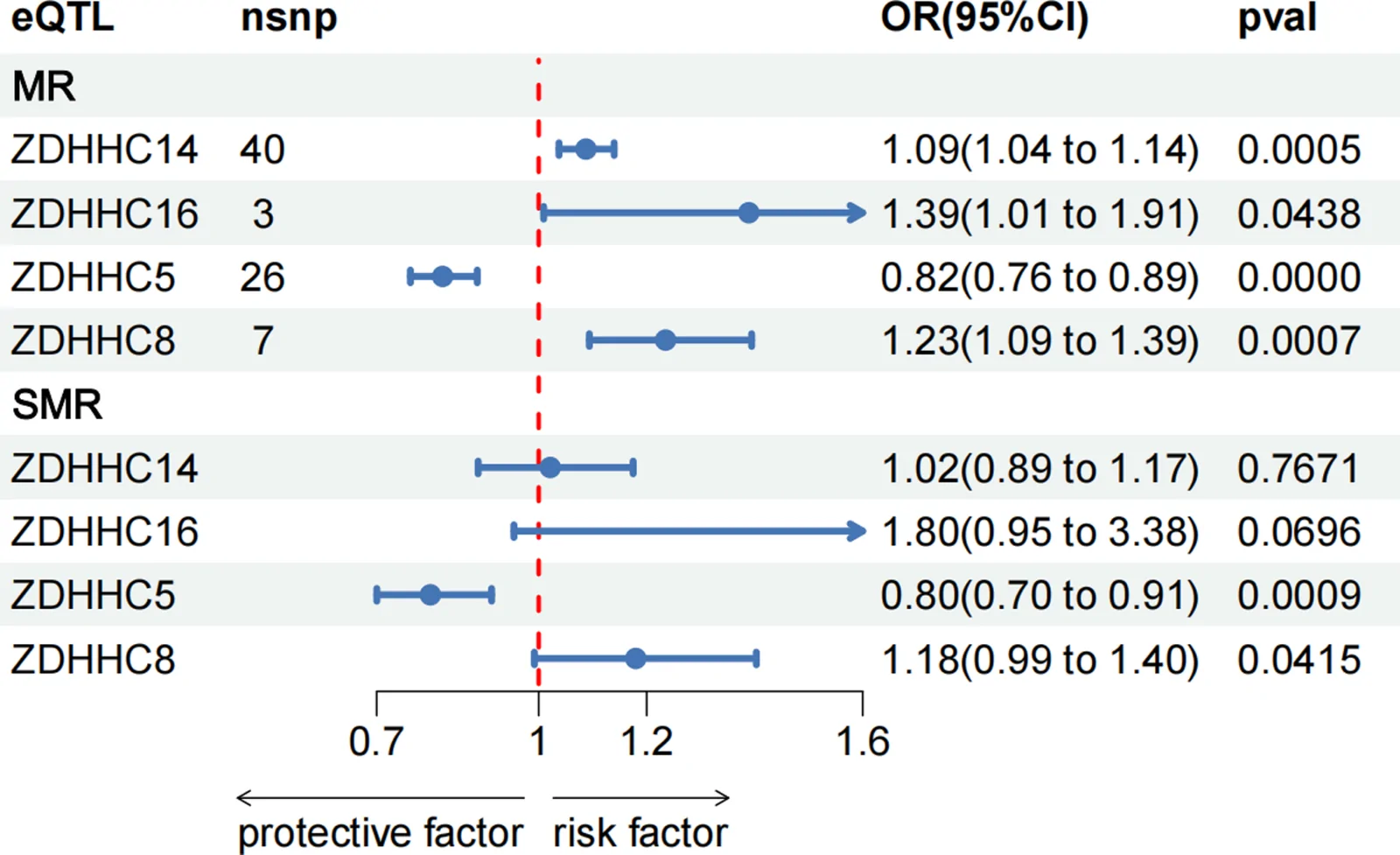
Forest plot of MR estimates for palmitoylation-related gene expression and osteoporosis risk. CI = confidence interval, eQTL = expression quantitative trait locus, MR = Mendelian randomization, nsnp = number of single-nucleotide polymorphism, OR = odds ratio, SMR = summary-data-based Mendelian randomization.

In SMR, ZDHHC5 remained inversely associated with osteoporosis (OR = 0.80, 95% CI: 0.70–0.91, *P* = .0009), and ZDHHC8 showed additional nominal evidence of association (*P* = .0415) (Fig. 3). SMR and HEIDI were used as supportive locus-level analyses rather than as independent proof of causality.

**Figure 3.**
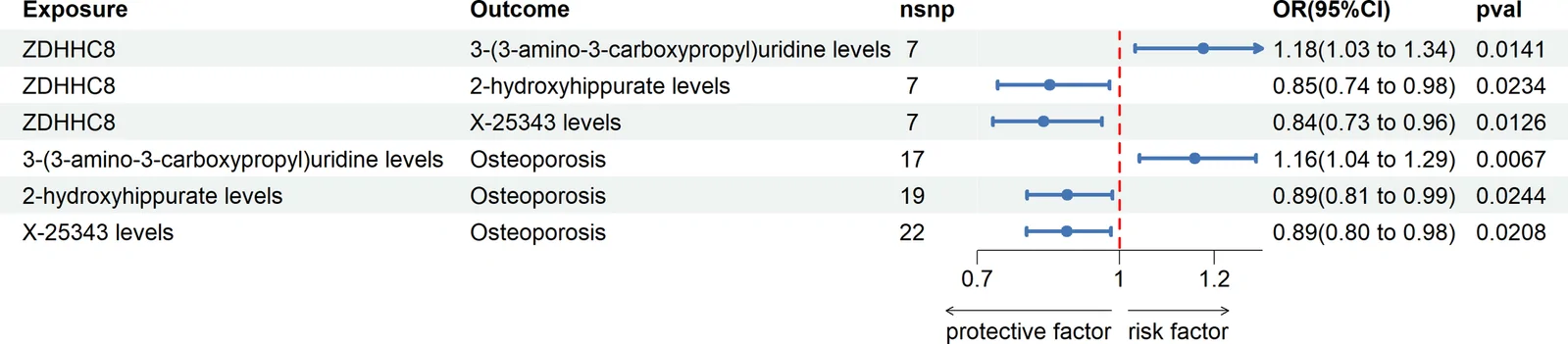
Summary of ZDHHC8-to-metabolite and metabolite-to-osteoporosis MR estimates used in the exploratory 2-step mediation analysis. CI = confidence interval, MR = Mendelian randomization, nsnp = number of single-nucleotide polymorphism, OR = odds ratio, ZDHHC8 = zinc finger DHHC-type palmitoyltransferase 8.

### 3.2. Analysis of differential expression in the GEO database

In GEO BMSCs, ZDHHC8 expression was higher in the osteoporosis group than in the non-osteoporotic comparison groups (reported *P* < .0001; Fig. 4). This direction was consistent with the MR finding. However, GSE35956 and GSE35958 share the same 5 osteoporosis donors, and GSE35959 is their SuperSeries. The expression result was therefore interpreted as supportive evidence from a small observational dataset, not as 3 independent replications.

**Figure 4.**
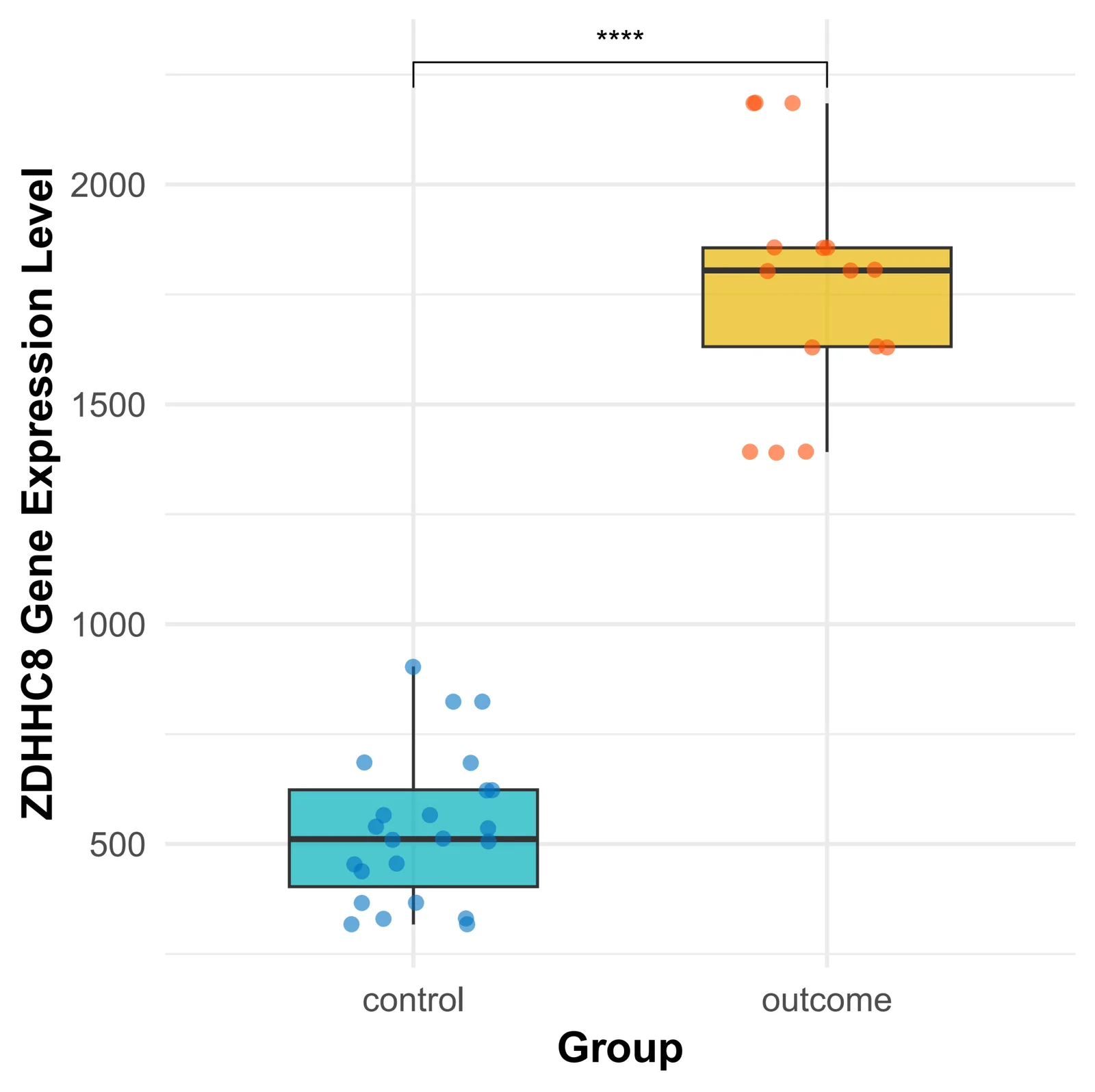
ZDHHC8 expression in human BMSCs from osteoporosis and non-osteoporosis groups. GSE35956 and GSE35958 contain overlapping osteoporosis donors; GSE35959 is the corresponding SuperSeries and is not an independent replication cohort. BMSC = bone marrow mesenchymal stem cell, ZDHHC8 = zinc finger DHHC-type palmitoyltransferase 8.

### 3.3. Mediation analysis

Two-step MR identified 3 metabolite traits with nominally significant and directionally coherent paths between ZDHHC8 and osteoporosis (Supplementary Material 2, Supplemental Digital Content 3). Because these signals did not meet the Bonferroni threshold for 1400 metabolite tests, they are reported as exploratory candidate mediators.

Higher genetically predicted ZDHHC8 expression was associated with higher 3-(3-amino-3-carboxypropyl)uridine (acp3U) levels (OR = 1.18, 95% CI: 1.03–1.34, *P* = .0141). Higher acp3U was also associated with higher osteoporosis risk (OR = 1.16, 95% CI: 1.04–1.29, *P* = .0067). The product-of-coefficients estimate was 0.0239 (1 = 0.1630; 2 = 0.1468), corresponding to an estimated mediated proportion of 11% and a direct-effect estimate of 0.1871.

Higher genetically predicted ZDHHC8 expression was associated with lower 2-hydroxyhippurate (OR = 0.85, 95% CI: 0.74–0.98, *P* = .0234) and lower X-25343 (OR = 0.84, 95% CI: 0.73–0.96, *P* = .0126). Higher levels of both metabolites were inversely associated with osteoporosis (OR is approximately 0.89 for each). Because both steps had negative coefficients, their products were positive: 0.0185 for 2-hydroxyhippurate and 0.0205 for X-25343. The estimated mediated proportions were 9% and 10%, respectively (Table 1). These estimates suggest possible pathways through loss of protective metabolite signals, but they should not be interpreted as confirmed mediation.

**Table 1:** 1400 metabolites act as mediators to mediate the mediating effects of ZDHHC8 and osteoporosis.

Gene	Intermediaries	Outcome	beta_all	beta1	beta2	beta12	beta_dir	beta12_p
ZDHHC8	3-(3-amino-3-carboxypropyl)uridine levels	Osteoporosis	0.2111	0.1630	0.1468	0.0239	0.1871	11%
ZDHHC8	2-hydroxyhippurate levels	Osteoporosis	0.2111	−0.1592	−0.1159	0.0185	0.1926	9%
ZDHHC8	X-25343 levels	Osteoporosis	0.2111	−0.1735	-0.1179	0.0205	0.1906	10%

ZDHHC8 = zinc finger DHHC-type palmitoyltransferase 8.

### 3.4. Sensitivity analyses

Sensitivity analyses are summarized in Supplementary Material 3, Supplemental Digital Content 2. The principal estimates were compared across IVW, weighted-median, and MR-Egger methods, and Cochran *Q* and MR-Egger intercept tests were used to assess heterogeneity and directional pleiotropy. Overall, the sensitivity analyses did not identify a clear contradiction to the main ZDHHC8 estimate. However, these tests have limited power when few instruments are available, and a nonsignificant result cannot exclude horizontal pleiotropy. We therefore describe the findings as genetically supported associations rather than definitive causal effects.

## 4. Discussion

This study found that higher genetically predicted ZDHHC8 expression was associated with a higher osteoporosis risk. The ZDHHC8 signal passed the Bonferroni threshold in the 22-gene MR screen, showed additional nominal support in SMR, and was directionally consistent with higher expression in osteoporosis-derived BMSCs. These complementary analyses strengthen gene prioritization; they do not, however, establish that ZDHHC8 is already a validated therapeutic target.

ZDHHC8 is a zinc finger DHHC-domain-containing S-acyltransferase, not an E3 ubiquitin ligase. DHHC enzymes catalyze protein S-palmitoylation and can change substrate localization, stability, and signaling.^[8–10]^ This distinction is important for biological interpretation. Recent studies have linked ZDHHC8 to palmitoylation-dependent control of ferroptosis-related proteins, including solute carrier family 7 member 11.^[29]^ A 2025 report also proposed glutathione peroxidase 4 (GPX4) as a ZDHHC8 substrate,^[30]^ although that work should be interpreted cautiously and independently validated. Another 2025 study identified ZDHHC20 as a GPX4 palmitoyltransferase, showing that GPX4 palmitoylation can regulate ferroptosis sensitivity.^[31]^ Together, these data support a broader connection between S-palmitoylation and ferroptosis but do not yet define the relevant ZDHHC8 substrate in bone.

A possible link between ZDHHC8 and osteoporosis is therefore ferroptosis and redox control. Ferroptosis is driven by iron-dependent lipid peroxidation, and GPX4 is a major suppressor of this process.^[32,33]^ Oxidative stress and ferroptosis have been implicated in impaired osteoblast function and bone loss.^[34–36]^ It is plausible that altered ZDHHC8 activity could affect the stability or localization of palmitoylated proteins that regulate redox balance in osteoblasts, osteoclasts, or BMSCs. This remains a mechanistic hypothesis. The present MR analysis cannot identify the relevant protein substrate, cell type, or direction of enzyme activity in bone tissue.

The metabolite analysis provides additional hypotheses. acp3U is a conserved RNA modification found in transfer RNA and is involved in RNA structure and translation-related processes.^[37–39]^ A direct role of circulating acp3U in bone remodeling has not been demonstrated. Its association with osteoporosis may reflect nucleotide turnover, RNA metabolism, renal handling, or another correlated pathway. Therefore, acp3U should be viewed as a candidate marker or intermediate pathway rather than a proven effector of ZDHHC8 in bone.

The second candidate, 2-hydroxyhippurate, should not be confused with “2-hydroxymauric acid,” as stated in the previous version. Hippurate-related metabolites can reflect host-microbial co-metabolism and systemic metabolic health.^[40]^ However, direct experimental evidence linking 2-hydroxyhippurate to osteoblast or osteoclast biology is currently limited. X-25343 is chemically unidentified in the source metabolomics resource, so mechanistic interpretation is even more limited. These uncertainties are an important limitation of using circulating metabolite traits as mediators.

Other palmitoylation-related genes also deserve attention. ZDHHC14 and ZDHHC5 passed the Bonferroni threshold in the primary gene screen, and ZDHHC5 also showed an SMR association. ZDHHC16 was nominally associated but did not survive gene-level multiple-testing correction. We focused on ZDHHC8 because it showed the most coherent combination of MR, SMR, and BMSC expression evidence, not because other DHHC enzymes are irrelevant. Future studies should compare these genes directly in osteoblast, osteoclast, and BMSC models and map their bone-specific substrates.

Several limitations should be considered. First, MR reduces conventional confounding and reverse causation but does not remove bias from weak instruments, horizontal pleiotropy, population stratification, assortative mating, or linkage disequilibrium. The relaxed *P* < 5 × 10^−^ instrument threshold used for some exposures may increase weak-instrument risk despite excluding *F* < 10 variants. Second, eQTL instruments were derived from blood, whereas osteoporosis is a bone and marrow disorder; tissue-specific regulation may differ. Third, the FinnGen outcome is a registry-based diagnosis phenotype. It does not provide a uniform DXA measurement for every participant, so diagnostic misclassification is possible. Fourth, the GWAS analyses were mainly restricted to European ancestry. Effect sizes may differ in Asian, African, admixed, younger, male, or premenopausal populations, and the findings should not be generalized without replication. Fifth, the GEO datasets are small and related. The osteoporosis donors overlap between GSE35956 and GSE35958, and GSE35959 is a SuperSeries rather than an independent cohort. Age, sex, fracture status, osteoarthritis or hip dysplasia in controls, cell passage, treatment history, and other clinical factors can affect gene expression. Thus, GEO cannot exclude confounding or reverse causation.

Sixth, the mediation analysis has additional assumptions. Serum or plasma metabolite measurements are influenced by diet, renal function, medication, microbiome, fasting state, pre-analytical handling, and platform measurement error. Genetically predicted metabolite levels may represent lifelong differences and are not equivalent to short-term pharmacological manipulation. The product-of-coefficients method also assumes compatible scales and no important exposure-mediator interaction. Most importantly, the 3 reported metabolite signals were nominal and did not pass Bonferroni correction for 1400 traits. They should therefore be considered candidate pathways requiring replication, not confirmed mediators.

Seventh, gene-environment interactions could modify the observed effects but could not be tested with the available summary statistics. Vitamin D and calcium status, smoking, alcohol use, physical activity, menopausal status, glucocorticoid exposure, anti-osteoporosis therapy, and other environmental or clinical factors may interact with bone-remodeling pathways. Future studies with individual-level data should test these interactions and perform sex- and age-stratified analyses.

Eighth, alternative explanations remain possible. The ZDHHC8 association could reflect another gene or regulatory element in linkage disequilibrium, a pleiotropic pathway unrelated to palmitoylation, or a blood eQTL signal that does not represent bone tissue. SMR/HEIDI and sensitivity analyses reduce some of these concerns but cannot eliminate them. Colocalization in bone-relevant tissues, fine mapping, clustered regularly interspaced short palindromic repeats perturbation, acyl-proteomics, and rescue experiments are needed to establish mechanism.

The clinical implications are therefore preliminary. These data do not justify ZDHHC8 inhibition, genetic screening, or metabolite supplementation in patients with osteoporosis. Palmitoylation is widespread and cell-type dependent, so systemic manipulation could have off-target effects. The immediate value of the study is target prioritization: ZDHHC8 can now be tested in bone-cell and animal models, while candidate metabolite pathways can be validated with targeted metabolomics. If the mechanism is confirmed, later work could examine tissue-selective modulation and whether ZDHHC8 adds information beyond established fracture-risk and bone mineral density measures.

From a public-health perspective, the findings do not change current prevention or treatment recommendations. Established measures such as fracture-risk assessment, adequate calcium and vitamin D status when clinically indicated, physical activity, fall prevention, and evidence-based anti-osteoporosis treatment remain central.^[41]^ The contribution of this work is mechanistic: it identifies a testable palmitoylation-related pathway that may help future studies of osteoporosis biology.

In summary, the study supports an association between genetically predicted ZDHHC8 expression and osteoporosis risk and provides convergent but not definitive evidence from SMR and BMSC expression data. The candidate mediation results involving acp3U, 2-hydroxyhippurate, and X-25343 are exploratory. Replication across ancestries, bone-specific eQTL or colocalization analyses, and direct functional experiments are required before ZDHHC8 can be considered a therapeutic target.

## Author contributions

**Conceptualization:** Wenli Yang.

**Data curation:** Wenli Yang, Shangyi Xu.

**Formal analysis:** Wenli Yang, Shangyi Xu.

**Funding acquisition:** Wenli Yang.

**Visualization:** Kaiwen Yang, Shuang Yang.

**Writing – original draft:** Wenli Yang.

**Writing – review & editing:** Shangyi Xu, Shuang Yang.






